# Utility of repeat cytological assessment of thyroid nodules initially classified as benign: clinical insights from multidisciplinary care in an Irish tertiary referral centre

**DOI:** 10.1186/s12902-016-0125-7

**Published:** 2016-08-02

**Authors:** Nigel Glynn, Mark J. Hannon, Sarah Lewis, Patrick Hillery, Mohammed Al-Mousa, Arnold D. K. Hill, Frank Keeling, Martina Morrin, Christopher J. Thompson, Diarmuid Smith, Derval Royston, Mary Leader, Amar Agha

**Affiliations:** 1 Department of Endocrinology, Beaumont Hospital and RCSI Medical School, Dublin 9, Ireland; 2 Department of Surgery, Beaumont Hospital and RCSI Medical School, Dublin 9, Ireland; 3 Department Radiology, Beaumont Hospital and RCSI Medical School, Dublin 9, Ireland; 4 Department of Pathology, Beaumont Hospital and RCSI Medical School, Dublin 9, Ireland

**Keywords:** Thyroid nodule, Fine needle aspiration, Thyroid cytology

## Abstract

**Background:**

Fine needle aspiration biopsy (FNAB) is the tool of choice for evaluating thyroid nodules with the majority classified as benign following initial assessment. However, concern remains about false negative results and some guidelines have recommended routine repeat aspirates. We aimed to assess the utility of routine repeat FNAB for nodules classified as benign on initial biopsy and to examine the impact of establishing a multidisciplinary team for the care of these patients.

**Methods:**

We performed a retrospective review of 400 consecutive patients (413 nodules) who underwent FNAB of a thyroid nodule at our hospital between July 2008 and July 2011. Data recorded included demographic, clinical, histological and radiological variables.

**Results:**

Three hundred and fifty seven patients (89 %) were female. Median follow-up was 5.5 years. Two hundred and fifty eight (63 %) nodules were diagnosed as benign. The rate of routine repeat biopsy increased significantly over the time course of the study (p for trend = 0.012). Nine Thy 2 nodules were classified differently on the basis of routine repeat biopsy; one patient was classified as malignant on repeat biopsy and was diagnosed with papillary thyroid carcinoma. Eight were classified as a follicular lesions on repeat biopsy—six diagnosed as benign following lobectomy; two declined lobectomy and were followed radiologically with no nodule size increase.

**Conclusions:**

The false negative rate of an initial benign cytology result, from a thyroid nodule aspirate, is low. In the setting of an experienced multidisciplinary thyroid team, routine repeat aspiration is not justified.

## Background

Thyroid nodules are palpable in 5 % of the population in iodine sufficient regions and in a much higher percentage in iodine deficient countries [1, 2]. However, neck ultrasound (US) identifies nodules in almost 40 % of the population [3]. The majority of nodules are benign with malignancy diagnosed in approximately 5–12 % of ultrasound detected nodules [4, 5].

An increasing number of thyroid nodules are incidentally detected by various modalities of imaging of the neck and thorax and contribute to an increased workload for endocrinologists, pathologists and radiologists. Several international societies have produced multidisciplinary guidelines for the assessment and management of thyroid nodules [6–9]. The overall objective of clinicians is to diagnose malignancy pre-operatively in patients with thyroid carcinoma and to limit unnecessary surgery in the vast majority with benign nodules. Neck ultrasound and fine-needle aspiration biopsy (FNAB) are promoted as the tools of choice for diagnosis.

Nodules classified as “benign” on cytology, following FNAB, account for the greatest proportion of cases seen in routine clinical practice [10]. However, cytological assessment of samples is somewhat subjective with several factors influencing the diagnostic outcome including the experience of the cytopathologist, as well as the adequacy of the sample [11, 12]. The negative predictive value of a “benign” cytology result is difficult to define and concern exists over false negative (falsely reassuring) results typically ranging from 1–11 %, potentially resulting in missed cancer diagnosis [13–18]. All guidelines recommend clinical follow-up for this group of patients. However, debate exists regarding the need for repeat cytological aspiration for nodules initially classified as benign [6].

## Methods

The primary aim of the study was to evaluate the utility of routine repeat FNAB for nodules initially classified as benign. Secondary aims were to determine the distribution of cytological diagnostic categories among a large cohort of unselected patients with thyroid nodules and to examine the effect of a multidisciplinary team on the assessment and care pathway of patients with thyroid nodules.

We performed a retrospective review of 400 consecutive patients (413 nodules) who underwent FNAB of a thyroid nodule at our hospital between July 2008 and July 2011. Thirteen patients had assessment of more than one nodule.

Patients were identified from the Department of Pathology database (WINPATH). All FNA biopsy results were reported according to the Royal College of Pathologists Thy Classification System (Table 1) [19]. Nodules were aspirated routinely if they were greater than 1.5 cm in diameter with any solid component or if they were less than 1.5 cm in diameter with suspicious clinical or sonographic features. Data recorded include demographic, clinical, cytological and radiological variables.

**Table 1:** Cytological classification following first FNAB [19]

Thy category	Description	No. of biopsies (%)
Thy 1	Inadequate/non-diagnostic	63 (15)
Thy 2	Benign	258 (63)
Thy 3	Follicular lesion	75 (18)
Thy 4	Suspicious for malignancy	8 (2)
Thy 5	Malignant	9 (2)

Data were collected by retrospective chart review of patients receiving routine clinical care and follow-up. All patients gave informed consent for FNAB in the context their standard clinical care. No further measures were taken beyond those of routine clinical practice. Data collection was undertaken via clinical audit by physicians caring for the patients; formal review and approval by the Research Ethics Committee was not required.

The first year of the study (July 2008–June 2009 inclusive) preceded the establishment of a formal multidisciplinary team for the management of thyroid nodules. During this period, thyroid aspirates were reported in a descriptive fashion rather than being stratified according to a cytological classification system. For all nodules evaluated during this period, the original slides were reviewed by a single cytopathologist and a Thy grade was assigned. Prospective reporting of thyroid aspirates according to the Thy classification system was implemented after June 2009.

A multidisciplinary team (MDT) for the management of thyroid nodules and thyroid cancer was established in July 2009 including specialists from thyroidology, endocrine surgery, histocytopathology, radiology, chemical pathology and radiation oncology. In addition to the routine reporting of Thy grade on cytology samples, the MDT developed an agreed protocol for the assessment of thyroid nodules based on the guidelines of the British Thyroid Association: 2007 Update (5).

The initial cytology category assigned to each nodule informed the management and surveillance as follows:

- Thy 1 (insufficient sample)—cases were discussed by the MDT and follow up involved repeat biopsy, surgical resection or clinical/radiological follow-up based on the risk profile and patient preference.
- Thy 2 (benign)—Routine repeat FNAB, after 6 months, was recommended to exclude a false negative result.
- Thy 3 (atypia or follicular lesion)—A lobectomy was offered for formal histological diagnosis
- Thy 4 (suspicious for malignancy)—Thyroidectomy was recommended due to the high risk of malignancy.
- Thy 5 (malignant)—Thyroidectomy was recommended due to the very high risk of malignancy.

Eighty nine percent of aspirates were performed under US guidance. The MDT met twice monthly during the study period to discuss both routine and complex cases.

### Statistics

Mean and standard error of the mean (SEM) were determined for normally distributed continuous data and median (standard deviation or range) was used for data not normally distributed.

The study period was divided into quartiles. MDT performance indicators were assessed in each quarter and trend analysis was performed using Chi square test. Statistical analysis was performed using GraphPad Prism 5 software (GraphPad Inc., La Jolla, CA, 2010).

## Results

Three hundred and fifty seven patients (89 %) were female. Median age was 54 years (range 16–89). Median nodule size (measured on ultrasound in 217 cases) was 25 mm in maximum transverse diameter. Only seven nodules were less than 10 mm in size. Median follow-up was 5.5 years (range 4.4–7.9).

Two hundred and thirty nine (58 %) nodules underwent a single biopsy; 156 (38 %) had a second biopsy. 14(3 %) and 4(1 %) underwent a third and fourth biopsy respectively. The distribution of initial Thy category is outlined in Table 1.

### Nodules with benign cytology (Thy2)

**Fig. 1:**
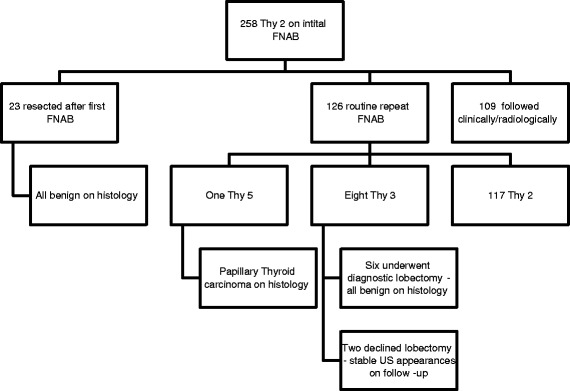
Routine management of patients with a thyroid nodule initially classified as Thy 2(benign)

**Fig. 2:**
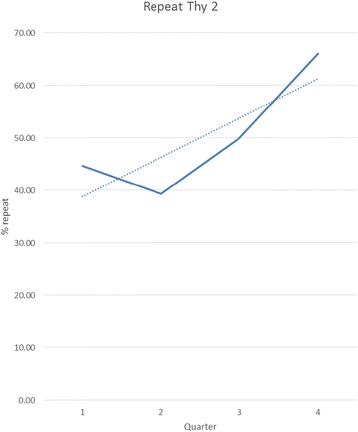
Trend (dotted line) for repeat FNA after initial aspirate classified as Thy 2 (benign); p for trend = 0.012. Solid line represents the absolute percentage of aspirates, initially classified as Thy 2, which were repeated in each quarter of the study period

Overall, 258 nodules (63 %) were initially classified as Thy 2 (benign). Management of patients with a nodule initially classified as Thy 2 (benign) is outlined in Fig. 1. Twenty three patients (9 %) elected, subsequently, to have surgery rather than conservative follow-up (lobectomy or thyroidectomy), principally due to local compressive symptoms (subjective or objective) or for cosmetic reasons—all were diagnosed as benign nodules. Among the remainder of this patient group, 126/235 (54 %) underwent routine repeat biopsy. The rate of routine repeat biopsy increased significantly over the time course of the study; p for trend = 0.012 (Fig. 2).

Nine Thy 2 nodules were classified differently following routine repeat biopsy (Fig. 1, Table 2). One patient was classified as Thy 5 (malignant) on repeat FNAB and was diagnosed with a solitary 3 cm papillary thyroid carcinoma (PTC) following a thyroidectomy—the entire nodule was malignant; the lesion had classical PTC morphology in some areas and in others demonstrated a Warthin-like tumour appearance. Two level VI cervical lymph nodes contained metastatic PTC - (Stage 1, pT2N1aM0). Eight were classified as Thy 3 after repeat aspiration; six underwent thyroid lobectomy and were diagnosed as benign; two declined lobectomy—they were followed with surveillance ultrasound and the nodules remained stable in size during 3 year follow up.

**Table 2:** Final cytological category of 126 nodules, initially classified as Thy2 (benign) after routine repeat FNAB

Result of routine 2^nd^ FNAB	No. of nodules (%)
Thy 1	0 (0)
Thy 2	117 (92.8)
Thy 3	8 (6.4)
Thy 4	0 (0)
Thy 5	1 (0.8)

The majority of patients with a nodule initially classified as Thy 2, who did not have surgery or a repeat biopsy, were followed for between 4–8 years without clinical or sonographic evidence of significant nodule enlargement. Therefore, the negative predictive value (NPV) of a Thy 2 biopsy was greater than 99 %; only one of 258 nodules, initially classified as benign, was subsequently found to harbor malignancy. If only biopsies which were repeated or followed by surgical resection are considered (*n* = 149), the NPV of an initial Thy 2 biopsy still exceeds 99 %.

### Nodules with other cytological classifications

Use of ultrasound guidance was significantly higher among sufficient/diagnostic (Thy2-Thy5) category aspirates in comparison with non-diagnostic aspirates (94 % versus 76 % of aspirates respectively). Therefore, lack of ultrasound guidance was strongly associated with insufficient biopsy material (*p* value < 0.0001).

Sixty three nodules (15 %) were initially classified as Thy 1 (insufficient). Twenty seven (43 %), of the nodules initially classified as Thy1, were predominantly or partially cystic in nature. Nineteen percent (12/63) of those initially classified as Thy 1 underwent surgical resection while 52 % (33/63) were re-biopsied. In the former group (surgically resected nodules), 4/12 patients were diagnosed with a malignant lesion. Repeat biopsy of a nodule originally classified as Thy 1 yielded a diagnostic sample in 57 %. Among those patients with two consecutive biopsies classified as Thy 1 (*n* = 12), four underwent surgery and 1 of the 4 had a malignant lesion diagnosed −2.7 cm locally invasive papillary thyroid carcinoma. The remaining patients, with either one or two insufficient cytology samples (*n* = 28), were followed clinically and/or radiologically and did not develop any concerning features to warrant resection.

**Fig. 3:**
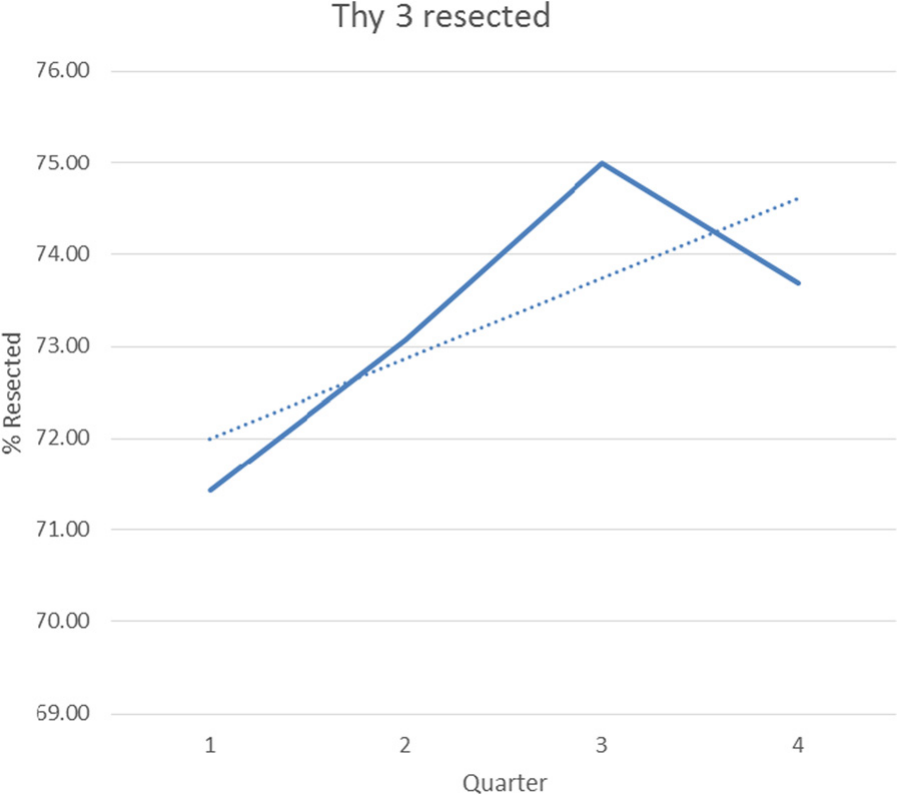
Trend (dotted line) for lobectomy following Thy 3 aspirate; p for trend 0.026. Solid line represents the absolute percentage of nodules classified as Thy 3 which were surgically resected during each quarter of the study period

Seventy five (18 %) of nodules were assigned to the Thy 3 category (atypia, follicular lesion or follicular neoplasm). The majority of Thy 3 nodules (55/75) were surgically excised; 29 % (16 nodules) were ultimately diagnosed as malignant. The rate of lobectomy for Thy 3 nodules increased significantly over the course of the study—p for trend 0.026 (Fig. 3). The remaining 20 patients have been followed with serial ultrasound—no nodule has increased significantly in size or developed any new suspicious radiological features.

Eight (2 %) nodules were initially classified as Thy 4 (suspicious for malignancy)—all proceeded to surgical resection. One patient was diagnosed with medullary thyroid carcinoma. Among the remainder, one patient was diagnosed with stage IV papillary thyroid carcinoma (PTC) while all others had stage I PTC. Thy 5 (malignant) nodules accounted for 2 % of cases (9 nodules). All had malignancy diagnosed following thyroidectomy—all patients had stage I differentiated thyroid cancer.

## Discussion

We describe the cytological findings and outcome in a large, consecutive series of patients who underwent diagnostic evaluation of thyroid nodules. The gender distribution and nodule size in our cohort are comparable to other large case series. However, length of follow up in our cohort is considerably longer than many studies in this field.

In keeping with previous research, most nodules in our cohort were classified as benign (Thy 2) after cytological assessment [10]. There is on-going debate, internationally, about the appropriate follow up of such cases. Guidance varies between repeat clinical, ultrasound and/or cytological assessment [6–9]. During the time course of this study, we adopted the contemporary recommendations of The British Thyroid Association to perform a routine repeat aspiration of lesions initially classified as benign (Thy 2) to rule out a false negative result [6].

Among the Thy 2 nodules in our cohort, 7 % had a different classification on repeat biopsy. However, amongst 126 patients who had a repeat FNAB, only one patient (0.8 %) initially classified Thy 2 was ultimately diagnosed with differentiated papillary carcinoma. Considering all nodules initially categorised as Thy 2 or only those who underwent repeat biopsy or surgery, the negative predictive value (NPV) of an initial benign cytology assessment exceeded 99 %. The majority of patients with benign cytology findings do not proceed to thyroidectomy and, given the indolent nature of many differentiated thyroid malignancies, there are concerns that a short follow up interval may lead to a verification bias and consequent overestimate of the NPV. In our study, only 23 (9 %) patients with initial benign cytology underwent surgical resection; however the long follow up (range 4–8 years) gives more credence to our conclusions.

Advocates of routine repeat FNAB of benign thyroid nodules on initial cytology voice concerns about false negative results and missed diagnoses of thyroid cancer. False negative rates, following a benign biopsy result, have been reported to be as high as 11 % in some historical series [17]. This led some authors to conclude that repeat aspiration is useful to reduce the rate of false negative results [17, 20, 21]. However, much of this data precede the widespread use of ultrasound-guided FNA resulting in a better cytological yield. In addition, cytology results were, previously, often not reported according to a validated classification system. Furthermore, repeat biopsies were not undertaken on a routine basis but, rather, were performed on the basis of clinical suspicion in many cases, leading to a somewhat biased study cohort.

Hamburger et al., in one of the few studies to perform routine repeat aspiration of benign thyroid nodules (not guided by ultrasound), reported that 9 % were assigned a different classification on repeat biopsy; 3 % were diagnosed with malignancy following thyroidectomy [13]. More recent research by *Singh Ospina* et al. reported that 1.2 % of nodules were classified as malignant when the FNAB was repeated after an initial benign cytological assessment-334 nodules, initially classified as benign, underwent repeat aspiration over a 10 year period; however, the authors do not report what proportion of initially benign biopsies were repeated [14]. Higher false negative biopsy rates-approximately 2.5 %—have been described in other recent retrospective studies [15, 22]. However, re-biopsy was undertaken on a selective basis, based on clinical or radiological concern during follow up, as opposed to the routine policy favoured in our study. Recent research has more accurately defined the natural history of nodules initially classified as benign; in a study of over 2000 such nodules, followed for over 8 years, there were no deaths due to thyroid cancer; thyroidectomy was undertaken in 24 % and the false negative rate was estimated at 1.3 % [18].

Enhanced resolution of ultrasound scanners has improved risk stratification of thyroid nodules. Several ultrasound features (e.g. irregular border, intranodular vascularity, hypoechoic echotexture, microcalcifications) have been shown to be more common in malignant thyroid lesions [23]. Ultrasound characteristics, combined with clinical features and specific risk factors can be used to risk stratify patients and inform the decision about the need for repeat biopsy [24, 25]. This approach is now advocated by many international experts and professional societies, who have suggested that routine repeat biopsy of nodules classified as Thy 2 (benign) may not be necessary in all cases [7]. Alternatively, nodules could be risk assessed according to the patient’s history as well as clinical and ultrasound features. Low risk lesions may not require biopsy. Furthermore, the size of the lesion is now recognised as a less important independent determinant of risk, unless the lesion has increased in size [26].

Multidisciplinary care is advocated for all patients with thyroid nodules and cancer [7]. However, there is a paucity of clinical data evaluating this approach to the care of this subgroup of patients [10]. Our local MDT was established towards the beginning of the study period and the temporal trends allow some assessment of its impact on patient care.

There was a high rate of Thy 1 (non-diagnostic/inadequate) aspirates in our series. Previous research has shown the value of US guidance in attaining a diagnostic sample [27]. Lack of US guidance in our study was strongly associated a non-diagnostic aspirate. Our rate of routine repeat biopsy increased significantly throughout the duration of the study (Fig. 1). This temporal trend supports the notion of increased integration of the MDT over time with improved implementation of a shared-care protocol.

Cytology has been shown to be inaccurate in distinquishing benign and malignant follicular lesions—ie Thy 3 lesions. International guidelines advise diagnostic lobectomy in such cases with predominantly follicular features (as opposed to those with only atypical cellular features) [7]. Our rate of lobectomy for Thy 3 lesions increased significantly throughout the study period—Fig. 2. We believe this may due to the establishment of the MDT which promoted a protocolisedapproach to care in line with international guidelines. The malignancy rate in Thy3 nodules in our study is somewhat higher than other series. However, only 75 % of our Thy 3 nodules were excised and this sample may be biased due to clinical and/or radiological concern about patients sent for surgery. Long-term clinical follow-up of non-operated Thy 3 nodules has not raised any concern or led to a recommendation for surgery.

Thy 4 category (suspicious for malignancy) only accounted for 2 % of nodules. However, all cases were diagnosed with malignancy after thyroidectomy. This may represent an over-reliance on this category as similar studies report malignancy rates between 60–80 % for this cytological category [10, 28, 29]. However, the number of Thy 5 (malignant) lesions is in keeping with international literature with no false positive cases. This emphasises the positive predictive value of fine-needle aspiration in the preoperative diagnosis of thyroid cancer.

This study supports and extends the findings of similar studies in this field. Very few previous studies have examined the impact of a routine repeat FNAB in patients with a thyroid nodule initially classified as benign, regardless of the clinical or radiological features. Our data support a very high accuracy of an initial, well-targeted FNAB with only 7 % of nodules receiving a different classification on re-biopsy and <1 % diagnosed with malignancy. The long follow up of our cohort adds further weight to our findings.

This study is limited mainly by the retrospective design. There may be a selection bias in the patients that had repeat biopsy, influenced by clinician and patient opinion at the time of evaluation but this is unlikely to have resulted in an underestimation of false negative results, as one would expect that if selection bias was a factor, it would have resulted in more diagnoses of thyroid carcinoma on repeat FNAB. In addition, greater detail of the ultrasound characteristics of nodules would have permitted analysis of these features for risk stratification of nodules. Finally, the study reports routine practice in a tertiary referral centre and the results may be challenging to reproduce in centres with a smaller case load, less complex case mix and where a similar, broad-based multidisciplinary team is not available.

## Conclusion

We conclude that the risk of malignancy in thyroid nodules initially classified as Thy 2 (benign), by an experienced cytopathologist, is very low when the FNAB is well-targeted and cases are managed by a MDT. Repeat biopsy is not necessary in all patients but could be reserved for selected cases with high risk clinical and/or radiological features.

In addition, the establishment of a multidisciplinary team, for the care of patients with thyroid nodules, is associated with improved implementation of international guidelines.

## Abbreviations

FNAB, fine needle aspiration biopsy; MDT, multidisciplinary team; US, ultrasound; NPV, negative predictive value; SEM, standard error of mean; SD, standard deviation
